# Healing Treatments in COVID-19 Patients: A Narrative Review

**DOI:** 10.3390/jcm12144672

**Published:** 2023-07-14

**Authors:** Thibault Sixt, Florian Moretto, Clementine Esteve, Michel Duong, Marielle Buisson, Sophie Mahy, Mathieu Blot, Lionel Piroth

**Affiliations:** 1 Infectious Diseases Department, Dijon-Bourgogne University Hospital, 21000 Dijon, France; florian.moretto@chu-dijon.fr (F.M.); clementine.esteve@chu-dijon.fr (C.E.); michel.duong@chu-dijon.fr (M.D.); marielle.buisson@chu-dijon.fr (M.B.); sophie.mahy@chu-dijon.fr (S.M.); mathieu.blot@chu-dijon.fr (M.B.); lionel.piroth@chu-dijon.fr (L.P.); 2 CHU Dijon-Bourgogne, INSERM, Université de Bourgogne, CIC 1432, Module Épidémiologie Clinique, 21000 Dijon, France; 3 Lipness Team, INSERM Research Centre LNC-UMR1231 and LabEx LipSTIC, University of Burgundy, 21078 Dijon, France

**Keywords:** SARS-CoV-2, COVID-19, drug, antiviral treatment, anti-inflammatory treatment, monoclonal antibodies

## Abstract

Since December 2019, many drugs have been evaluated or advocated as potential treatments of SARS-CoV-2 induced disease (COVID-19), including many repositioned drugs and some others specifically developed for these diseases. They can be roughly classified into three categories according to their main mechanism of action (passive immunization, direct antivirals, and anti-inflammatory treatments), and their use depends on the stage of the disease. Despite often promising preclinical data, most of the treatments evaluated failed to show a significant clinical benefit. In addition, a few others have seen their effectiveness affected by the occurrence of SARS-CoV-2 variants and sub-variants. Herein, the aim of this article is to take stock of the data available as of the 14th of July 2022, concerning the specific healing options evaluated for patients suffering from COVID-19. We focus particularly on healing treatments of COVID-19 and do not deal with preventive treatments such as vaccine. Associated therapies such as venous thromboembolism prophylaxis are not detailed since they are covered in a specific chapter of this issue. Passive immunization, especially through monoclonal antibodies, showed a positive impact on the clinical evolution, whether in outpatients or inpatients without oxygen supply. However, their effectiveness strongly depends on the type of SARS-CoV-2 variant, and often decreases or even vanishes with the most recent variants. Among direct antiviral treatments, ritonavir-boosted nirmatrelvir appears to currently be the cornerstone in the management of early infections, but its use may be limited by drug interactions. Remdesivir remains as an alternative in this situation, even though it is potentially less convenient. Anti-inflammatory treatments have often been shown to be the most effective in inpatients with oxygen supply. Dexamethasone is now a cornerstone of management of these patients. Added tocilizumab seems beneficial in the case of hyper inflammation. JAK inhibitors and anakinra have also gained an interest in some studies. As a conclusion of this narrative review, the best treatment strategy has yet to be defined and is likely to evolve in the future, not only because many other drugs are still under development and evaluation, but also because of the viral epidemics and epidemiology evolution.

## 1. Introduction

Since December 2019, the new coronavirus named Severe Acute Respiratory Syndrome Coronavirus 2 (SARS-CoV-2) has been responsible for the Coronavirus disease 2019 (COVID-19) pandemic [1], which had a dramatic impact on healthcare systems, global economy, and social life. 

SARS-CoV-2 is a single-stranded RNA-enveloped virus that invades cells through its spike protein by binding to the angiotensin-converting enzyme-2 (ACE-2) receptor. These receptors are present in the lung, kidney, heart, and brain. Then, a fusion of the viral envelope with the cellular membrane is performed and the virus hijacks the cell machinery. This first phenomenon corresponds to the viral and early phase of the disease [2].

Then, in some cases, this induces pyroptosis and the release of damage-associated molecular patterns (ATP, nucleic acid…), leading to inflammatory response involving pro-inflammatory cytokines and chemokines (IL-6, CXCL10, MCP-1…), followed by recruitment of myeloid cells into the lungs. The aim of this reaction is to eliminate the infected cells before the virus spreads, but sometimes (8 to 15 days after symptom onset) it leads to a hyper-inflammatory response and lung and multi-organ damage [2,3,4]. This second phenomenon corresponds to the inflammatory and late phase of the disease.

The clinical spectrum of COVID-19 is thus broad. Very often, the clinical presentation of COVID-19 is asymptomatic, or is mild to moderate with flu-like symptoms, due to the invasion and the replication of SARS-CoV-2 into host cells [5,6,7,8,9]. This corresponds to the viral and early phase of the disease.

Subsequently, some patient suffering from COVID-19 worsen and may develop acute respiratory distress syndrome with the need of mechanical ventilation in an intensive care unit [10,11,12]. This phenomenon corresponds to the late hyper-inflammatory response.

On the 14th of July 2022, 8072 trials on COVID-19 were ongoing or completed, [13]. Research was also accelerated, in particular through the use of platform trials where multiple interventions can be evaluated simultaneously against a common control group within a single master protocol [14].

**Figure 1:**
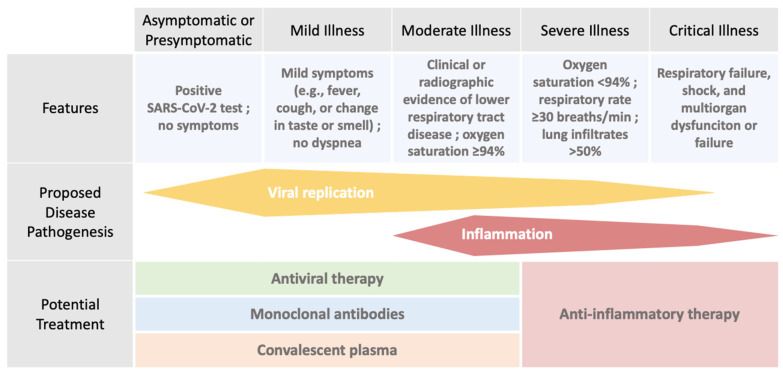
Management of COVID-19 according to Disease Stage or Severity, adapted from Rajesh T. Gandhi et al. [15].

These treatments can be classified in three categories: passive immunization, antiviral agents, and anti-inflammatory agents. All these treatments, according to their mechanisms of action, are summarized in Table 1. Their indication in the management depends on their mode of action as well as the phase of the disease (detailed in Figure 1).

Nevertheless, the results observed and the subsequent management of patients are challenged by the appearance of new variants, especially Omicron and its sub-variants, which influence the effectiveness of some treatments, in particular monoclonal antibodies.

Herein, the aim of this article is to provide an overview of the data available as of the 14th of July 2022, concerning the medical treatments evaluated for patients suffering from COVID-19.

This narrative review focuses only on healing treatments of COVID-19 and does not deal with preventive treatments such as vaccine. It also does not deal with associated therapies such as venous thromboembolism prophylaxis, which is covered in a specific chapter of this issue.

## 2. Materials and Methods

We used the electronic scientific resources PubMed, Google Scholar, Web of Science, and Science Direct to look for all relevant English articles employing the phrases “Treatment and Management” in connection with “COVID-19”. Articles concerning preventive treatment, vaccination, post-exposure treatment, and thromboembolic prophylaxis were excluded. Articles not written in English were also excluded. The authors manually checked the reference lists of the selected literature for completeness in order to validate the inclusion of information pertinent to the treatment of COVID-19. Finally, articles were included in this review if they follow the inclusion and exclusion criterion, were published in English, and if they were original research articles or reviews (resulting most of the time in a publication in a peer-reviewed journal).

## 3. Therapeutic Options in Early COVID-19 (Patient within the First Week of Symptoms and Requiring Oxygen Therapy)

At this phase of the disease, treatments with antiviral activity (either passive immunization or direct acting antiviral agents) are the main drugs of interest. 

For clarity, all these treatments are summarized in Table 2, with their indications.

### 3.1. Passive Immunization with Monoclonal Antibodies

Neutralizing antibodies can block viral entry by preventing the S protein from binding to host cell receptors (e.g., ACE2). Several monoclonal antibodies have been developed as prophylaxis or as an early treatment of COVID-19. They are administered parenterally, intramuscularly, or sometimes intravenously. However, their efficacy greatly varies depending on the SARS-CoV-2 variant. As a consequence, the virological evolution during the pandemics has led to abandonment of several of them due to lack of efficacy on the most recent variants.

Bamlanivimab and etesevimab are recombinant neutralizing human IgG1κ monoclonal antibodies directed against the spike protein of SARS-CoV-2. When the alpha variant was predominant, it demonstrated a 70% relative reduction in COVID-19-related hospitalization or death from any cause (*p* < 0.001) [16]. However, its effectiveness mostly vanished with the Delta variant [17], as well as with the recent omicron variant, regardless of the sub-variant BA.1 or BA.2 [18,19,20].

The casirivimab and imdevimab combination (RONAPREVE©) was associated with a 71.3% reduction in COVID-19-related of hospitalization or all cause death (*p* < 0.0001), still in alpha variant predominant period [21]. However the use of this monoclonal antibody cocktail has been largely limited by the emergence of the omicron variant and its sub-variants, on which its efficacy is greatly reduced [18,19,20].

Sotrovimab (XEVUDY©), a human monoclonal antibody, contains a two–amino acid Fc modification (termed LS) to increase half-life and potentially improve bioavailability in the respiratory mucosa. This modification may permit therapeutic concentrations for longer durations [22]. Sotrovimab has been shown to have potent effector functions in vitro that may result in immune-mediated viral clearance [23,24] demonstrated a 85% relative risk reduction in mortality or hospitalization (*p* = 0.002). Sotrovimab is effective to neutralize the BA.1 Omicron sub-variant [23]. However, the emergence of the BA.2, BA.4, and BA.5 sub-variants has significantly reduced its activity [19,20] and its use is no longer recommended [25].

#### Some Monoclonal Antibodies Remain of Clinical Interest by 14 July 2022

Tixagevimab and cilgavimab (EVUSHELD©) inhibit SARS-CoV-2 spike protein-directed attachment. Their efficacy was established in preventing the development of symptomatic COVID-19 [26]. The use of tixagevimab and cilgavimab (300/300 mg) was associated with a 50% reduction in the risk of developing severe COVID-19 or death (from any cause) compared to placebo in outpatients who had been symptomatic for seven days or less [27]. Its activity against the BA.1 Omicron sub variant is nevertheless reduced [18], and even further reduced against BA.2, precluding its use in such a situation. This decrease in efficacy is also observed, albeit to a lower extent, against BA.4 and BA.5, since cilgavimab (but not tixagevimab) still remains active [19,20]. This legitimates that the combination is currently an option in the BA.4/BA.5 epidemiological context, within the lack of other effective and available antibody.

Bebtelovimab is a fully human neutralizing IgG1 monoclonal antibody directed against the spike protein of SARS-CoV-2. In a recent study, its association with etesevimab and bamlanivimab was associated with greater viral clearance and a reduction in time to sustained symptom resolution [28]. Moreover, its efficacy is maintained against BA.4 and BA.5 subvariants [19,20], and thus bebtelovimab appears to so far be the best monoclonal antibody option against all variants [29]. However, clinical data are still scarce in July 2022, especially in high-risk patients. In addition, despite this promising efficacy, the manufacturer Eli Lilly has decided to supply doses of bebtelovimab solely to the US Government.

### 3.2. Passive Immunization with Convalescent Plasma

The effectiveness of convalescent plasma is based on the neutralizing action of the antibodies but is also based on antibody-dependent cellular cytotoxicity and on enhanced phagocytosis. Results regarding the efficacy of convalescent plasma as a treatment of early COVID-19 are discrepant. In an Argentinian trial, the early use of convalescent plasma (within 3 days of onset of symptoms) was associated with a reduction in the risk of severe respiratory disease (RR: 0.52; 95% CI [0.29–0.94]) compared to placebo [30]. Whereas this benefit was not observed in other trials [30,31,32,33], it was confirmed in a recent multicenter, double-blind, randomized, controlled trial including 1225 unvaccinated patients suffering from early COVID-19 (within 8 days of onset of symptoms). Convalescent plasma was associated with a relative risk reduction of 54% of COVID-19–related hospitalization within 28 days after transfusion [32]. However, the potential efficacy of convalescent plasma in vaccinated patients has not been studied yet. Furthermore, the dose (neutralizing antibody titers) of convalescent plasma remains to be defined. Finally, studies are difficult to compare because convalescent plasma has evolved with the appearance of new variants. Moreover, these limited beneficial effects are to be counterbalanced with possible side effects. Indeed, in a large study including more than 20,000 patients, 146 transfusion reactions were noted, mainly of cardiac decompensation [34]. Another concern with convalescent plasma is the risk of antibody-dependent enhancement. This phenomenon could exacerbate COVID-19 severity and is already well described for other respiratory viruses, such as respiratory syncytial virus and measles. While there is a theoretical risk that convalescent antibodies could enhance disease via antibody-dependent enhancement, this has not yet been described in the various studies on this therapy [35].

### 3.3. Direct Antiviral Agents

Nirmatrelvir boosted with ritonavir (PAXLOVID©) is currently the main direct antiviral treatment. Nirmatrelvir inhibits the 3-chymotrypsin like protease (3CLpro), also called the main protease (MPro). This prevents the assembly of viral particles and thus interrupts viral particle production. Despite frequent mutations in the viral genomes of SARS-CoV-2, nirmatrelvir is still active against recent coronavirus mutants, including Omicron subvariants [36,37]. However, when used alone, the in vivo median plasma concentrations of nirmatrelvir remained above the 90 efficacy concentration (EC90) threshold only between 2 and 4 h [38], due to rapid elimination. By slowing the metabolism of nirmatrelvir by inhibiting hepatic enzymes, ritonavir acts as a booster allowing nirmatrelvir concentrations to stay above the EC90 for 12 to 18 h [38]. The recommended treatment thus associates nirmatrelvir 300 mg and ritonavir 100 mg twice a day, orally, for five days. 

In the EPIC-HR phase 3 trial including more than 2000 patients, symptomatic, unvaccinated, non-hospitalized adults at high risk for progression to severe COVID-19, the use of nirmatrelvir/ritonavir started within the first 5 days after the symptoms onset was associated with a 88.9% relative risk reduction in hospitalization or death (*p* < 0.001) [39]. This benefit was also observed in a recent Israelian study conducted in real life settings and including 180,351 patients (75% of them correctly vaccinated). The use of PAXLOVID™ was associated with a significant decrease in the rate of severe COVID-19 or mortality (HR of 0.54 95% CI, 0.39–0.75) [40].

However, the use of nirmatrelvir/ritonavir may be limited in clinical practice due to drug–drug interactions, especially anti-rejection treatments but also hypolipidemic, antiarrhythmic or antipsychotic treatments. However, many of them can be stopped for 5 days and for some others, dose adjustment charts have been developed. 

Nevertheless, and despite this difficulty, nirmatrelvir/ritonavir is currently the first line recommended treatment in early COVID-19 in most international recommendations. Indeed, the recent IDSA recommendation advocate the use of nirmatrelvir/ritonavir within five days of symptom onset for ambulatory patients with mild to moderate COVID-19 at high risk for progression to severe disease [25] (Table 3).

Remdesivir is an alternative antiviral drug, with in vitro activity against some RNA viruses, including MERS-CoV, SARS-CoV-1 and 2 [41]. Remdesivir causes premature termination of viral RNA transcription. Its efficacy was rather disappointing in hospitalized patients for COVID-19, with limited or no significant effect [42,43].

By contrast, in outpatients with COVID-19 and at high risk for severe disease or death, the use of remdesivir (3-day course, 200 mg intravenously at day 1 and 100 mg per day at day 2 and 3) within 7 days of symptoms onset reduced the risk of hospitalization or death by 87% compared to placebo (HR: 0.13; 95% CI [0.03–0.59]; *p* = 0.008) [44]. Its use may be currently limited by its low availability in some places, in particular in France, as well by the need for intravenous administration over 3 days. However, remdesivir is a potential effective option during the early phase of the disease when ritonavir-boosted nirmatrelvir (PAXLOVID™) may not be administered due to drug interaction. The IDSA recommend the use of remdesivir within seven days of symptom onset among patients (ambulatory or hospitalized) with mild-to-moderate COVID-19 at high risk for progression to severe disease [25] (Table 3).

Molnupiravir (LAGEVRIO©) is a small-molecule ribonucleoside prodrug of N-hydroxycytidine (NHC), effective against SARS-CoV-2 and other RNA viruses [45]. This treatment leads to an accumulation of lethal errors throughout the viral genome of SARS-CoV-2 that ultimately render the virus noninfectious and unable to replicate [46]. Molnupiravir seemed to be promising in phase 1 and 2 trials. However, in the recent MOVe-OUT trial, a phase 3 randomized trial, a significant discrepancy between the data collected over the first period (interim analysis) of the study and those collected over the second period (difference between the interim analysis and the final analysis) was observed. Indeed, during the first period 7.3% of patients receiving molnupiravir compared to 14% patients receiving placebo were dead or hospitalized until day 28, as compared to, respectively, 6.2% versus 4.7% in the second period [47]. Moreover, the reduction in the risk of progression to COVID -19 with molnupiravir was approximately 30%, lower than that described with monoclonal antibodies (approximately 80%). With the loss of effectiveness of certain monoclonal antibodies, the use of molnupiravir may be subject to further discussion. For the moment, the French National Authority for Health (HAS) denied early access to molnupiravir, and the IDSA recommend the use of molnupiravir within five days of symptom onset only for ambulatory patients (≥18 years) with mild to moderate COVID-19 at high risk for progression to severe disease who have no other treatment options [25] (Table 3).

### 3.4. Other Drugs (Miscellaneous)

Several drugs (most of them repurposed) with variable supposed mechanisms of activity have been advocated to have a potential interest in early COVID. Despite a few reported preliminary positive clinical results, methodological questioning and the scarcity of the data do not currently provide sufficient evidence to recommend the use of these drugs.

Out of them, the efficacy of fluvoxamine, a selective serotonin reuptake inhibitor and a σ-1 receptor agonist, has been assessed in the TOGETHER trial [48]. The use of fluvoxamine within 7 days of symptom onset in outpatients at risk for severe COVID-19 was associated with a reduced risk of hospitalization (or stay at the ER for more than 6 h) for COVID-19 (RR:0.68; 95% Bayesian credible interval (95% BCI): [0.52–0.88]), but several methodological uncertainties and the lack of confirmation in other trials do not allow confidence about this potential efficacy. Thus, the IDSA recommended the use of fluvoxamine only in the context of a clinical trial, and this drug is currently not recommended in other international guidelines [25].

Nitazoxamide has been positioned in the past as a treatment of diarrhea caused by *Cryptosporidium parvum* and *Giardia intestinalis* because of an in vitro activity against MERS and certain animal coronaviruses, as well as a potential anti-inflammatory effect [49]. Its efficacy was assessed in a study in patients suffering from mild or moderate COVID-19. The use of nitazoxamide within 72 h of symptom onset tended to be associated with a relative risk reduction in progression to severe disease of 85% in the key secondary analysis (*p* = 0.07) [50]. The lack of final results and independent confirmation precludes its use in clinical practice.

Anti-inflammatory drugs, which were shown to be effective in late COVID, as we will see, did not demonstrate any interest in early COVID. This is particularly the case for corticosteroids, which were shown even more to be deleterious in the RECOVERY trial when used at an early stage of COVID-19. Indeed, the benefit on survival was particularly marked for patients requiring ventilatory support whereas for patients without oxygen had a higher risk of mortality (RR 1.27; 95% CI 1.00–1.61) [51].

## 4. Therapeutic Options in Late COVID-19 (Hospitalized Patients for COVID-19 Requiring Oxygen Therapy)

For clarity, all these treatments are summarized in Table 4, with their indications.

Dexamethasone (DXM) was the first treatment shown to be effective during COVID-19. In the RECOVERY platform trial [51], 2104 patients were randomly assigned to receive dexamethasone (6 mg/day once daily for up to 10 days or until hospital discharge if sooner) and 4321 were to receive usual care alone. The use of dexamethasone resulted in lower 28-day mortality (22.9% vs. 25.7%, RR: 0.83 95% CI [0.75–0.93]; *p* < 0.001) and a higher level of discharge from hospital within 28 days (67.2% vs. 63.5%, RR 1.10 95% CI [1.03–1.17]). In a prospective meta-analysis, including 1703 participants with 678 participants (40%) in the corticosteroid group (systemic dexamethasone, hydrocortisone, or methylprednisolone) and 1025 participants (60%) in the usual care or placebo group, a significant association lower 28-day mortality with DXM was observed (RR: 0.64 CI95% [0.5–0.82]; three trials), but not with hydrocortisone (RR 0.69 CI95% [0.43–1.12]; (3 trials), or prednisone (RR 0.91 CI95% [0.29–2.87] (1 trial)). This benefit is however dependent on the clinical severity. In a recent trial, the use of a higher dexamethasone dose (12 mg) was not associated with a better prognosis at 28 days (adjusted RR 0.86 [99% CI, 0.68–1.08]) [52]. This phenomenon is also highlighted in a large meta-analysis summarizing the effect of corticosteroids used at different dosages [53].

Thus, dexamethasone (6 mg per day during 10 days) is strongly recommended by the IDSA [25] and ESCMID [54] for patients with hypoxemia related to this viral infection and requiring oxygen therapy.

Tocilizumab (ROACTEMRA©) was considered since compelling data suggested that IL-6 could be a key cytokine in COVID-19 pathophysiology, and that drugs targeting IL-6 such as tocilizumab are an appealing therapeutic strategy [55,56]. In the RECOVERY platform trial including 4116 patients, where tocilizumab was administrated for participants having a demonstrated clinical evidence of progressive COVID-19 (which was defined as <92% oxygen saturation on room air or receiving oxygen and C-reactive protein (CRP) ≥ 75 mg/L) and compared to standard of care, a 28-day mortality of 29% was observed for patients receiving tocilizumab as compared to 33% for patients receiving SOC (RR 0.86; 95% CI [0.77–0.96]) [57]. In a prospective WHO meta-analysis, including 19 trials corresponding to 8048 patients, the use of tocilizumab was associated with a reduction in 28-day mortality compared with usual care or placebo (OR: 0.83; 95% CI [0.74–0.92]) [58]. Thus, both the IDSA [25] and the ESCMID [21] recommend the use of tocilizumab among hospitalized adults with progressive severe (meaning needing oxygen) or critical COVID-19 (meaning patient patients on mechanical ventilation, on ECMO, or having end organ dysfunction as is seen in sepsis/septic shock), and who have elevated systemic inflammation markers (CRP > 75 mg/L).

Tofacitinib (XELJANZ©) is a JAK inhibitor that preferentially inhibits JAK-1 and JAK-3. Its use induces an inhibition of the inflammatory cascade that results in systemic inflammation in patients with severe COVID-19. In the STOP-COVID trial [59], a double-blind, randomized, placebo-controlled, multi-center study comparing tofacitinib versus standard of care and including 289 patients hospitalized for COVID-19, tofacitinib was associated with a lower risk of the composite outcome of death or respiratory failure compared to no tofacitinib (RR 0.63; 95% CI [0.41, 0.97]). However, no effect was observed on mortality alone (RR: 0.49; 95% CI [0.15, 1.63]) or progression to mechanical ventilation or ECMO by day 28 (RR 0.25; 95% CI [0.03, 2.20]). The IDSA recommended the use of tofacitinib in hospitalized adults with severe (meaning needing oxygen) COVID-19, but not on non-invasive or invasive mechanical ventilation. This treatment has to be associated with at least prophylactic dose anticoagulant and patients should not receive tocilizumab or other IL-6 inhibitors in association. On the other hand, tofacitinib is not mentioned in the ESCMID recommendation [54].

Baricitinib (OLUMIANT©) is a selective Janus kinase 1 and 2 (JAK1 and JAK2, respectively) inhibitor. As with tofacitinib, Baricitinib may decrease cytokine-mediated inflammation induced by the SARS-CoV-2. Baricitinib may also have an antiviral action by disrupting the AP2-associated protein kinase 1 (AAK1), which is a key regulators of endocytosis of SARS-CoV-2 [60]. In the COV-BARRIER trial, a phase 3, double-blind, randomized, placebo-controlled trial including 1525 participants, baracitinib was associated with a 38.2% relative reduction in the 28-day all-cause mortality (RR 0.57; 95% CI [0.41–0.78]). Moreover, the 60-day all-cause mortality was 10% (*n* = 79) for baricitinib and 15% (*n* = 116) for placebo (RR 0.62; 95% CI [0.47–0.83]. In a meta-analysis, allocation to baricitinib or other JAK inhibitor was associated with a 20% proportional reduction in mortality (rate ratio 0.80; 95% CI [0.71–0.89]) [61]. Thus, the IDSA [25] recommended the use of baracitinib associated to standard of care among hospitalized adults with severe COVID-19. By contrast, and as for tofacitinib, baricitinib is not mentioned in the ESCMID recommendation [54].

Anakinra (KINERET©) is a recombinant human IL-1 receptor antagonist. Its efficacy was first evaluated in CORIMUNO-ANA-1 [62], a French, multicenter, open-label, randomized clinical trial. In this trial, including 116 participants, anakinra did not improve the coprimary outcomes that were proportional to patients who had died or needed noninvasive or mechanical ventilation (corresponding to a World Health Organization Clinical Progression Scale (WHO-CPS) score of >5 points) at D4, and survival with no need for mechanical ventilation or noninvasive ventilation at D14 (RR: 0.97, 90% CI [0.62–1.52]). This study was stopped early for futility. 

In a more recent study, the SAVE-MORE trial, a double-blind, randomized controlled trial evaluated the efficacy and safety of anakinra for 594 patients suffering from COVID-19 at risk of progressing to respiratory failure. Patients were defined as at risk when soluble urokinase plasminogen receptor plasma (suPAR) levels were ≥6 ng/mL, since early elevations in suPAR are associated with a risk of progression to severe respiratory failure or death [63]. The adjusted proportional odds of having a worse clinical status (assessed by the 11-point WHO-CPS) with anakinra, as compared to placebo, was 0.36 (95% CI 0.26–0.50).

The problem is that this dosage of suPAR is often unavailable in current practice. Despite this, the French High Authority for Health authorized the use of anakinra during COVID-19. However, this treatment is not mentioned in the American or European recommendations [25,54].

Beside these anti-inflammatory drugs, most antiviral drugs failed to show a benefit in late COVID-19. However, sabizabulin, a microtubule disruptor has shown promising results. This treatment has a direct antiviral effect but also an anti-inflammatory effect by inhibiting polymerization of tubules in cells and thus not only cellular trafficking of SARS-CoV-2 but also the tubule-triggering of the innate inflammatory response. This both anti-inflammatory and antiviral drug may be promising in late COVID-19, as the recent interim results of a phase 3 trial were associated with a 55.2% relative reduction in deaths compared with placebo (*p* = 0.0042) [64].

## 5. Main Treatments Which Failed to Show a Benefit and Are Not Recommended in the Management of COVID-19

Since the beginning of COVID-19, many treatments failed to show significant clinical efficacy. In this section we report the most supported and mediatic treatments, with strong clinical experimental evidence.

Hydroxychloroquine (HCQ): Hydroxychloroquine has shown antiviral properties against many different viruses, including coronaviruses [65]. More than twenty randomized trials including more than 1000 patients have assessed the effect of hydroxychloroquine. In particular, both the two major platform trials (RECOVERY trial [66] and SOLIDARITY trial [42]) failed to show an impact on day-28 all-cause mortality (RR 1.06, 95% CI [0.97–1.16], and RR 1.19, 95% CI [0.89–1.59], respectively), as well as on intermediate clinical markers. This was also the case in a Cochrane meta-analysis [67] (8040 participants in nine trials, RR of death 1.09; 95% CI [0.99–1.19]). 

Lopinavir/ritonavir: Lopinavir, which also demonstrated in vitro inhibition of SARS-CoV-1 and MERSCoV replication [68], had no impact on day-28 all-cause mortality in the RECOVERY and in the DISCOVERY trials (5040 participants, RR 1.03 95% CI [0.91–1.17]) [69], and 2791 patients RR 1.00; 95% CI [0.79–1.25], respectively) [42], and was often associated with gastrointestinal side-effects leading to early withdraw [70].

Azithromycin: Despite its antiviral activity in vitro against ribonucleic acid viruses [71], in addition to its anti-inflammatory activity [72], no benefit was observed with azithromycin in the RECOVERY trial (2582 hospitalized patients, 28-day mortality, RR 0.97; 95% CI [0.87–1.07] [73]), or in the COALITION II open-label randomized trial [74]. 

Ivermectin: Also advocated to be active in vitro against some viruses, including SARS-CoV-2 [75], in addition to anti-inflammatory effects, ivermectin had no impact on all-cause mortality (RR: 0.37; 95%CI [0.12–1.13]; 425 participants; five trials), or on length of stay (mean difference: 0.72; 95%CI [−0.862–2.29]; 176 participants; three trials) in a recent meta-analysis [76].

Colchicine: This anti-inflammatory drug, which inhibits neutrophil chemotaxis and inflammasome signaling [77], tended to reduce the risk of hospitalization or death (Odds Ratio (OR) 0.79; 95% CI [0.61–1.03]; *p* = 0.08) in a trial [78], but failed to confirm any efficacy in the RECOVERY trial (28-day all-cause mortality RR 1.01, 95% CI [0.93–1.10]) [79]. 

Interferon beta-1a: As with lopinavir, interferon beta-1a demonstrated in vitro inhibition of SARS-CoV-1 and MERSCoV replication [68]. However, its use was not associated with a reduced mortality in the SOLIDARITY trial [42] (459 participants, 28-day mortality, RR 1.16, 95% CI [0.96–1.39]).

## 6. Discussion

In 2 years, many drugs have been evaluated as potential treatments in COVID-19, with a wide variety in the size and quality of the studies conducted. In this narrative review we aimed to make a review of the available data, but many drugs are still under evaluation and this narrative review cannot be completely exhaustive. Nevertheless, the suggested potential efficacy of many candidate drugs could be rather quickly ruled out (e.g., hydroxychloroquine, azithromycin, and ivermectin). 

On the other hand, several therapies were found to be effective, with efficacy depending not only on the stage of COVID-19, but also on the epidemiological context (i.e., SARS-CoV-2 variant involved). 

In early COVID-19, antiviral treatments are the reference. Nirmatrelvir boosted with ritonavir (PAXLOVID™) should be considered as the first-line therapy, but its use may be limited by drug–drug interactions. Remdesivir may be an option when nirmatrelvir/ritonavir cannot be used but is less convenient to use because of a parenteral administration route for 3 days, and its lack of available everywhere. So far, it can be advocated that the efficacy of antivirals remains stable whichever the SARS-CoV-2 involved. However, it cannot be ruled out that viral resistance to these drugs with clinical and public health significance could emerge in the future. Combination strategies thus may be an option to prevent this risk, even though clinical trials are currently missing regarding such strategies.

This may be particularly relevant since monoclonal antibodies, which also have an antiviral activity and were very promising during the first waves, have been greatly affected by the appearance of variants, as recently observed in particular with the Omicron BA.4 and BA.5 sub-variants. As a result, only the association of tixagevimab and cilgavimab (EVUSHELD©) at high doses is expected to remain effective with the latter subvariants. Bebtelozumab, which appears to be the most interesting option in vitro, whatever the variant considered, will be likely not available outside of the USA [80]. Convalescent plasma‘s benefit remains debatable, considering the divergent results currently available, and needs confirmation.

In late COVID-19, when oxygen support is needed, most of the studies highlighted the role of dexamethasone as a cornerstone in COVID-19 drug management. Tocilizumab and JAK inhibitors (in association with dexamethasone) are the second pillars of care in this case, when patients need oxygen and are likely to be used in patients with a high inflammatory syndrome. Other anti-inflammatory treatments have shown their interest, in particular anakinra, but their use and place in the therapeutic arsenal has to be confirmed.

Despite all these data, we must keep in mind that the evaluation of effectiveness always has to be interpreted and relativized with the context of their realization. Many results are drawn from platform trials (RECOVERY, SOLIDARITY, etc.) using standard of care as a comparator (but not placebo). In addition, these platform studies include all types of patients, and we cannot exclude the efficacy of some therapies in specific populations. For instance, some groups of immunocompromised patients may experience more benefit from passive immunotherapy given underlying B and T cell immune deficiencies, and/or antiviral therapies because of the prolonged excretion of the virus.

Furthermore, most of the studies with available results mainly included patients seronegative for COVID-19, and particularly non-vaccinated people. It can thus be advocated that the “relative” benefit (in terms of risk reduction) observed would be the same in non-vaccinated as in vaccinated people, but that the “absolute” benefit would be lower. Another key point is that the involved variants of concern are no longer the same as those involved at the time most of these studies were conducted. All in all, both variants types and vaccination-related immunity probably modify the pathophysiology of the disease, in particular with a decrease in the risk of progression to the inflammatory phase, leading to a reduction in mortality and hospitalizations [81,82].

This narrative review has several limitations. Like all narrative reviews, this work does not use explicit and systematic criteria for the search and critical analysis of the literature. While we have made conscious efforts to ensure that the studies selected are high powered and representative, selection bias may have occurred during the data extraction. Moreover, the pandemic is still in progress, new therapeutics could be developed after the 14 of July 2022. On top of that, certain therapeutics assessed at that time may have lost their effectiveness with the appearance of new variants.

Future perspectives concerning the management of COVID-19 will be impacted by the occurrence of a new variant which will impose the need for a renewal of monoclonal antibodies. Each of the currently recommended antivirals has drawbacks (paxlovid has a large number of drug–drug interactions, remdesivir is administered intravenously) and future antiviral treatments will need to be more malleable. The Japanese company Shionogi has already communicated on promising preliminary results on a new protease inhibitor [83]. Finally, future perspective may rely on combined drugs. Their objective is to attack the virus in different ways, in order to be more quickly effective, but also to prevent the occurrence of resistance. The other interest is to target different phases of the disease, for example, using direct antiviral treatments to target the viral phase with anti-inflammatory treatments to target the inflammatory phase.

In conclusion, the best treatment strategy is likely to evolve in the future, not only because many drugs are still under development and evaluation, but also because of the evolution of this context [84]. All these notions of treatment are likely to change in the months and years to come with the contribution of ongoing studies, but also with the possibility of new variants and new vaccine strategies, for which we must be attentive and adapt accordingly.

## Figures and Tables

**Table 1:** Classification of treatments of interest in COVID 19 according to their mode of action.

Antiviral Effect	Anti-Inflammatory Effect	Passive Immunization
Remdesivir	Corticosteroids	Bamlanivimab and etesevimab
Molnupinavir (LAGEVRIO©)	Janus Kinase (JAK) inhibitors (Tofacitinib, Baricitinib)	Casirivimab and indevimab (RONAPREVE©)
Nirmatrelvir boosted with ritonavir (PAXLOVID©)	Anakinra (KINERET©)	Sotrovimab (XEVUDY©)
Sabizabulin		Tixagevimab and cilgavimab (EVUSHELD©)
		Bebtelovimab
		Convalescent plasma

**Table 2:** Summary of treatments which are recommended or may be of interest in early COVID-19 in July 2022, with their indications.

	Recommended	May Be of Interest
Nirmatrelvir boosted with ritonavir (PAXLOVID©)	Mild or moderate symptoms Onset of the symptoms < 5 days Having at least one risk factor for severe COVID-19	
Remdesivir	Mild or moderate symptoms Onset of the symptoms < 5 days Having at least one risk factor for severe COVID-19	
Tixagevimab and Cilgavimab (EVUSHELD©)	Tixagevimab and cilgavimab 600 mg Mild or moderate symptoms Onset of the symptoms < 8 days Having at least one risk factor for severe COVID-19	
Molnupiravir (LAGEVRIO©)		Mild or moderate symptoms Onset of the symptoms < 5 days Having at least one risk factor for severe COVID-19
Convalescent plasma		Mild or moderate symptoms Onset of the symptoms < 8 days Having at least one risk factor for severe COVID-19
Bebtelovimab		Need to have precise efficacy against Omicron BA.2 BA.4 and BA.5 subvariant
Fluvoxamine		Mild or moderate symptoms onset of the symptoms < 7 days Having at least one risk factor for severe COVID-19
Nitazoxamide		Mild or moderate symptoms Onset of the symptoms < 3 days

**Table 3:** Risk factors for the progression to severe COVID-19 or hospitalization according to the Infectious Disease Society of America [25].

Older age (for example ≥65 years of age) Obesity or being overweight (for example, adults with BMI > 25 kg/m^2^, or if age 12–17, have BMI ≥ 85th percentile for their age and gender based on CDC growth charts) Pregnancy Chronic kidney disease Diabetes Immunosuppressive disease or immunosuppressive treatment Cardiovascular disease (including congenital heart disease) or hypertension Chronic lung diseases (for example, chronic obstructive pulmonary disease, asthma [moderate to severe], interstitial lung disease, cystic fibrosis and pulmonary hypertension) Sickle cell disease Neurodevelopmental disorders (for example, cerebral palsy) or other conditions that confer medical complexity (for example, genetic or metabolic syndromes and severe congenital anomalies) Having a medical-related technological dependence (for example, tracheostomy, gastrostomy, or positive pressure ventilation [not related to COVID-19])

**Table 4:** Summary of treatments which are recommended or may have an interest in late COVID 19 in July 2022, with their indications.

	Recommended	May be of Interest
Dexamethasone	6 mg/day during 10 days When oxygen is needed	
Tocilizumab (ROACTEMRA©)	When oxygen is needed and CRP > 75 mg/L	
Tofacitinib (XELJANZ©)	When oxygen is needed and Elevated inflammatory syndrome In the absence of non-invasive or invasive mechanical ventilation	
Baracitinib (OLUMIANT©)	When oxygen is needed and Elevated inflammatory syndrome	
Anakinra (KINERET©)		When oxygen is needed and If soluble urokinase plasminogen receptor plasma (suPAR) levels was ≥6 ng/mL
Sabizabulin		When oxygen is needed and High risk of acute respiratory distress syndrome (ARDS) and death

## Data Availability

Not applicable.
